# Catatonia and Mutism: Neurotic, Psychotic, or Organic Disorder?

**DOI:** 10.1155/2021/5936673

**Published:** 2021-10-28

**Authors:** Pilar de Jaime Ruiz, Jose Luis García-Fogeda Romero, Luis Gutiérrez-Rojas

**Affiliations:** ^1^Psychiatry Department, Hospital Clínico San Cecilio, Granada, Spain; ^2^Internal Medicine Department, Hospital Clínico San Cecilio, Granada, Spain; ^3^Department of Psychiatry, University of Granada, Granada, Spain

## Abstract

*Background*. Catatonia is caused by a variety of psychiatric and organic conditions. The onset, clinical profile, and response to treatment may vary depending on the underlying cause. Catatonia is more likely to be associated with neurotic and psychotic disorders, but some psychiatric symptoms are key components in the clinical presentation of other medical conditions. *Case Report*. We report the case of a woman who started showing paroxysmal recurrent episodes since the age of 57 years, characterized by surrounding disconnection, disorientation, and muscle spasm (myoclonus), followed by a postictal state. In the following months, the symptoms evolved to akinetic mutism, catatonia, and rapidly progressive vision and audition loss. She underwent a battery of tests, most of them inconclusive, until a neoplastic meningoencephalitis was diagnosed after more than two years of symptoms. Numerous medical conditions can mimic psychiatric disorders. This uncommon presentation may lead to a late diagnosis and treatment initiation, increasing significantly morbidity and mortality. A differential diagnosis with infectious, autoimmune, and neoplastic etiologies should always be carried out.

## 1. Introduction

Even though catatonia can be caused by a variety of psychiatric and organic conditions, ranging from neurologic to systemic diseases, it is more likely to be associated with neurotic and psychotic disorder [1]. Catatonia is found in 10% of psychiatric inpatients and is more common in patients with mood disorders, especially mania, than in patients with schizophrenia [2]. The diversity of symptoms often leads to a delay in diagnosis and treatment initiation, increasing morbidity and mortality significantly. Albeit catatonia and akinetic mutism are commonly related to psychiatric diseases, numerous medical conditions can mimic psychiatric disorders [3]. A differential diagnosis with infectious, autoimmune, and paraneoplastic encephalitis should always be carried out [4].

In this report, we describe the case of a patient who consulted due to a two-year history of paroxysmal recurrent episodes, with surrounding disconnection, disorientation, and muscle spasm (myoclonus), followed by a postictal state. Eventually, she ended up in a catatonic state, with rapidly progressive vision and audition loss and almost surrounding disconnection. The patient died seven months later.

The purpose of this report is to describe a case of psychomotor impairment and catatonia due to a neoplastic meningoencephalitis associated with an unknown primary tumor. We also aim to create a differential diagnosis, which should lead us to investigate other causes apart from mental illnesses, and to enumerate the general principles of a comprehensive assessment.

## 2. Case

The patient, Ms. A., was a 57-year-old married woman and mother to a son. She had no relevant medical history. As surgical background, she had undergone surgery of groin hernia, varicosities in lower limbs, appendectomy, and tonsillectomy. Ms. A. was an ex-smoker (who quitted at the age of 39 years). She had a family history of breast cancer in her mother and her sister.

As for her psychiatric history, she suffered from depressive mood after her father's death, when the patient was 49 years old. She was treated with individual support psychotherapy and venlafaxine 150 mg/day for one year, showing good response and clinical remission.

### 2.1. First Episodes

In March, 2015, Ms. A. attended the emergency room presenting with a confused state, spatial disorientation, inability to recognize her relatives, and a slurred speech. Due to the patient's mental condition, the information was provided by her family.

In July, 2015, Ms. A. had a new episode, this time characterized of hyperventilation and upper and lower intermittent shakes. The patient was conscious, and she linked the episode with some stressful familiar situations. In both these episodes, Ms. A. was assessed by a neurologist, who performed blood tests, a computed tomography (CT) scan, an electroencephalogram (EEG), a sleep-deprived EEG, and a brain magnetic resonance imaging (MRI) scan but no abnormalities were detected.

Both neurologist and psychiatrist assessed the patient. Since physical tests were unremarkable, Ms. A. was diagnosed initially with a conversion/dissociative disorder.

We referred Ms. A. to neurology and psychiatry departments in outpatient visits. After performing neurological and psychiatric assessments with the consent and collaboration of the patient and her relatives, she was started on an anticonvulsant (levetiracetam 1000 mg/day), an antidepressant (desvenlafaxine 50 mg/day), and a benzodiazepine (alprazolam sustained release tablets 1 mg/day).

### 2.2. New Episodes Two Years Later

Between July, 2015, and December, 2016, episodes as described above continued, but they were less frequent and self-limiting. From January, 2017, the episodes started to be more severe and disabling. In March 2017, Ms. A. presented to the hospital's emergency room by her relatives with a new episode of confusion, spatial disorientation, myoclonus seizures, and stereotypies (such as lip smacking), during which she remained unconscious for ten minutes. Afterwards, she recovered by herself, but drowsiness and spatial disorientation persisted. After recovering, when we tried to establish contact with Ms. A. and elucidate what had happened, she could not remember the episode. According to her relatives at that time, she was taking desvenlafaxine 50 mg/day, alprazolam sustain-released tablets 0.5 mg/day, and olanzapine 5 mg/day. In order to rule out any iatrogenic cause, we replaced alprazolam 0.5 mg/day with diazepam 5 mg/8 h (as a muscle relaxer) and quitted desvenlafaxine.

In April, 2017, Ms. A. started to display migraine-like symptoms, drowsiness, and being increasingly more excited, up to the point that she could barely breathe, suffering from trismus and emitting gibberish sounds. Furthermore, she also presented with muscle contractions and claw digits. In our physical examination, she was neither cooperative nor resistant, but she appeared to be indifferent. We requested additional tests in order to rule out other organic conditions. Brain CT scan, EEG, and pulmonary scan (in order to rule out a pulmonary thromboembolism), as well as laboratory test results, were unremarkable, again. A psychiatric orientation was then reassumed as a key component of this clinical profile. At that time, we added quetiapine 25 mg/8 h replacing olanzapine for the purpose of reducing a long-term anxious mental state, which was the patient's most remarkable clinical manifestation during those episodes.

### 2.3. Clinical Worsening and Hospitalization

In May 2017, within a few weeks, the patient developed hyperventilation, muscle stiffness, lockjaw, walking difficulty, falls, and psychiatric symptoms consisting on visual hallucinations, bizarre behaviour, and some abnormal gestures such as trying to grab nonexistent objects in the air. This initial presentation was followed by an acute course towards a catatonic state, mutism, rapidly progressive vision and audition loss (initially, she could not recognize colours, and then, she lost visual and audition acuity), frequent seizures, muscle rigidity, and facial dyskinesia. The patient was eventually admitted to a psychiatric inpatient unit, as our initial presumptive diagnosis had been, until that point, a mental disorder. There, she was started on intravenous diazepam 5 mg/8 h and haloperidol 5 mg/8 h. We did not observe any response, so we went further with physical evaluation.

**Figure 1:**
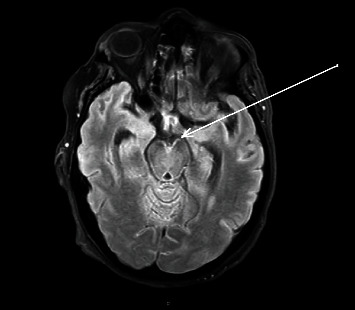
Brain MRI showing linear hyperintensities in basal segments and VIII, VII, V, and III cranial nerves (FLAIR sequence) (please see the arrow).

**Figure 2:**
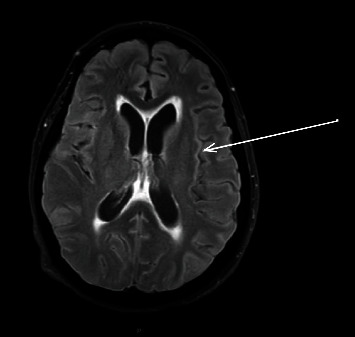
T2 sequence revealing hyperintensity in the walls of both lateral ventricles, as well as subtle communicant hydrocephalus (please see the arrow).

A new brain MRI scan was performed, showing linear hyperintensities in basal segments and the VIII, VII, V, and III cranial nerves in FLAIR sequence, in the walls of both lateral ventricles in T2 sequences, as well as subtle communicant hydrocephalus (see Figures 1 and 2). A lumbar puncture showed very high cerebrospinal fluid (CSF) pressure (55 mmHg, normal value 10-15 mmHg), notable hyperproteinorrachy (470 mg/dL, normal value 15-15 mg/dL), with normal glucose levels and cell account. Gram stain cultures and C-reactive protein (CRP) test for bacteria and viruses were also negative.

Eventually, CSF cytology detected tumor cells. These cells were compatible with a metastatic breast adenocarcinoma, according to immunohistochemical stain. However, we never found any primary tumor after performing body CT scan, mammography, and positron emission tomography- (PET-) CT. The clinical course was very aggressive, with refractory catatonia and rigidity, and the patient died few weeks later.

### 2.4. Final Diagnosis

The final diagnosis was carcinomatous meningoencephalitis, probable breast cancer as primary tumor.

## 3. Discussion

Catatonia is a neuropsychiatric syndrome easily identifiable by the clinician. However, an etiological diagnosis is not always easy. The *Diagnostic and Statistical Manual of Mental Disorders, 4th Edition, Text Revision (DSM-IV-TR)* [5] recognizes that many cases are secondary to an organic disorder, under the heading of catatonic disorder due to a general medical condition (CD-GMC). The fifth DSM edition (DSM-5) recognizes that catatonia can occur in the context of another mental disorder or as a disorder due to another medical condition. Despite the amount of evidence, there are not many publications available regarding characteristics of organic catatonia.

Many acute cases are attended at emergency departments, where the catatonias are often attributed to psychiatric problems, instead of medical and neurological conditions [1, 6]. This means delays in diagnosis and initial management, which are associated with increased morbidity and complications; some of these are serious and life-threatening. Many of these complications are related to decreased dietary and liquid intake and others to hospitalization and immobilization, such as rhabdomyolysis, deep vein thrombosis, pulmonary thromboembolism, and aspiration pneumonia, among others [6].

### 3.1. Differential Diagnosis

Apart from psychiatric disorders, catatonia is secondary to numerous medical and neurological conditions. In a cohort of patients from a single institution [7], no reports of any psychiatric condition and a previous history of seizures were found in a greater proportion in the group of CD-GMC. Nonetheless, in clinical practice, there is a great deal of overlap between neurological and psychiatric processes. Thus, our approach is to rule out organic disease always, even when mental illness is the main suspicion.

In the study cited above, 21% of the cases were attributable to CD-GMC [7]. Cortical processes and encephalitis were the most common etiologies. Encephalitis was also observed to be among the most common etiologies of CD-GMC in a systematic literature review [8]. On this line, anti-N-methyl-D-aspartate receptor (NMDAR) encephalitis might be the paradigm of CD-GMC [9]. Since it was identified just a little more than a decade ago, a lot of researches have been done; this rare condition receives a lot of interest among clinicians and investigators. It is believed that for many decades, anti-NMDAR encephalitis remained underdiagnosed, and a substantial number of patients were mistakenly categorized as having a psychiatric disorder. Even nowadays, distinguishing the disease from a primary psychiatric disorder is challenging.

The differential diagnosis of CD-GMC also includes other autoimmune and inflammatory mechanisms, as well as infectious, neoplastic, metabolic, toxic, and other causes.  Table 1 provides a comprehensive list of etiologies [1].

### 3.2. General Principles for Etiologic Assessment

After syndrome categorization, it is necessary to focus on etiological diagnosis. It is essential to make a comprehensive clinical history, including patient's past medical, surgical, psychiatric, and drug/toxic history and symptoms of the current episode. Most patients are not able to perform an interview due to their catatonic state, so questioning accompanying persons is recommended.

Considering physical and psychiatric examinations, catatonic signs and symptoms in patients with CD-GMC and in psychiatric patients are mostly indistinguishable [10]. This was the conclusion of a retrospective chart review of 47 cases, which found a slightly higher prevalence of negativism and higher frequency of echo phenomena in patients with CD-GMC, but in general with a similar distribution of catatonic signs regardless of the etiology.

The appropriate evaluation [1, 6] would consist of a basic blood test with biochemical and haematological parameters. Specific investigations, such us drug blood/urine screen, viral serology, or nuclear autoantibodies, will be done depending on the clinical history, symptoms, and subsequent results in initial tests. Neuroimaging is necessary to rule out structural abnormalities; MRI may be more sensitive than CT. EEG can identify abnormal epileptiform activity seen, which is mostly related to nonconvulsive epileptic status.

Lumbar puncture was found to be the most useful procedure to make final diagnosis in CD-GMC [7, 8]. Therefore, analysis of CSF, including biochemical parameters, cell count, culture, and cytology, may probably be obligatory in all circumstances.

### 3.3. Summary

In this case, the patient underwent a remarkable great number of tests; unfortunately, most of them were inconclusive. In fact, a first MRI was completely normal, and no abnormalities were found until a second MRI was performed two years later. Although prognosis of metastatic infiltration of CNS is bleak, a more rapid diagnosis may have been made if CSF had been analysed in an earlier stage.

This highlights how psychiatric symptoms often lead to a psychiatric diagnosis straightaway, with other possibilities seldom being considered. Although catatonia and akinetic mutism are commonly related to psychiatric diseases, several medical conditions can mimic psychiatric disorders [1, 5, 6, 10]. Cancer is just one of them, which can induce neurological and behavioural disturbances. Encephalitis is also an important differential diagnosis [9].

The correct diagnostic subcategorization of catatonia is critical for proper prognostication, identification of disease-specific acute therapies, and long-term therapeutic management of the underlying disease state. Delays are linked to poorer outcomes [6]. We strongly suggest to always rule out an organic condition as cause. We also recommend the use of lumbar puncture and CSF analyses for patients with catatonic disorder of unclear etiology [7, 8]. Focus should be placed on patients with neurological symptoms and recurrent bizarre episodes of undetermined origin with partial response to the treatment, as these patients may be at higher risk of any organic condition, and a complete differential diagnosis should be carried out.

## Figures and Tables

**Table 1:** Causes of catatonia (adapted from Jaimes-Albornoz and Serra-Mestres [1]).

*Psychiatric and neurodevelopmental* (i) Mania and depression (bipolar disorder), unipolar depression, late-onset depression, schizophrenia, and chronic psychoses (ii) Anxiety disorder, dissociative disorder and Ganser syndrome, adjustment disorders, acute stress reactions, obsessive-compulsive disorder, Prader–Willi syndrome, autistic spectrum disorders, and Gilles de la Tourette syndrome

*Neurological* (i) Cerebrovascular disease, haemorrhagic infarcts (ii) Neoplasms, paraneoplastic encephalopathy, and malignant and benign central nervous system tumors (iii) Encephalitis (including anti-NMDAR, herpes, human immunodeficiency virus (HIV), postimmunisation, and encephalitis lethargica), meningitis, and cerebral abscesses (iv) Postencephalitic states, especially with parkinsonism, progressive multifocal encephalopathy (v) Epilepsy (absence seizures, complex nonconvulsive partial seizures, generalised and complex partial (focal) status epilepticus, postictal states) (vi) Neurosyphilis, other central nervous system infections: typhoid fever, tuberculosis, borreliosis, malaria, trypanosomiasis, hidatidosis (vii) Parkinson's disease, Lewy body disease, frontotemporal dementia, Alzheimer's disease, vascular dementia, Creutzfeldt-Jakob disease, fatal familial insomnia (viii) Motor neuron disease, Wilson's disease, Huntington's disease, multiple sclerosis, progressive supranuclear palsy (ix) Brain trauma acute and squeal, Wernicke's encephalopathy, hepatic encephalopathy, central pontine myelinolysis (x) Insomnia, narcolepsy, Tay-Sachs disease, tuberous sclerosis (xi) Hydrocephalus

*Metabolic and endocrine, haematological, and immune* (i) Diabetic ketoacidosis, hypercalcemia, renal failure, liver failure (ii) Acute intermittent porphyria, homocystinuria, membranous glomerulonephritis, hyponatremia, hypernatremia (iii) Lysosomal disease, hypothyroidism, hyperthyroidism, hyperparathyroidism, hypoglycemia, Sheehan's syndrome (iv) Addison's disease, Cushing's disease, syndrome of inappropriate antidiuretic hormone secretion (SIADH) (v) Vitamin B12 deficiency, nicotinic acid deficiency, pellagra (vi) Systemic lupus erythematosus, pediatric autoimmune neuropsychiatric disorder associated with streptococcal infection (PANDAS) (vii) Antiphospholipid syndrome, renal and hepatic transplant, Langerhans carcinoma

*Pharmacological, toxic, and other* (i) Typical and atypical antipsychotics (use and withdrawal) including clozapine, levodopa, amantadine, serotonergic drugs (selective serotonin reuptake inhibitors (SSRIs), trazodone, venlafaxine, etc.), lithium, acetyl-cholinesterase inhibitors (ii) Cephalosporins, ciprofloxacin, levofloxacin, azithromycin, levetiracetam, sodium valproate, gabapentin (iii) Disulfiram, paracetamol, aspirin, tramadol, hydroxyzine, antiretroviral, adrenocorticotropic hormone (ACTH), steroids (iv) Cyclosporine, chlorphenamine, methylphenidate, morphine, methadone, meperidine, allopurinol (v) Benzodiazepine withdrawal, cocaine, cannabis, lysergic acid diethylamide (LSD), mescaline, ketamine, phencyclidine, amphetamines, organophosphates, ethylene, carbon monoxide, severe burns
